# Nursing experiences in specialized services in child and adolescent mental health: a systematic review of qualitative studies

**DOI:** 10.1590/0034-7167-2022-0550

**Published:** 2023-12-08

**Authors:** Priscilla de Oliveira Luz, Adriana Almeida de Souza, Gabriella de Andrade Boska, Marilia Mastrocolla de Almeida Cardoso, Bruna de Paula Candido, Márcia Aparecida Ferreira de Oliveira

**Affiliations:** I Universidade de São Paulo. São Paulo, São Paulo, Brazil; II Universidade Estadual Centro-Oeste. Guarapuava, Paraná, Brazil; III Faculdade de Medicina de Botucatu, Hospital das Clínicas. Botucatu, São Paulo, Brazil

**Keywords:** Nursing, Mental Health, Child, Adolescent, Systematic Review, Enfermería, Salud Mental, Niño, Adolescente, Revisión Sistemática, Enfermagem, Saúde Mental, Criança, Adolescente, Revisão Sistemática

## Abstract

**Objective:**

to synthesize evidence from qualitative studies on nursing experiences regarding child and adolescent mental health care in specialized services.

**Method:**

a systematic review with meta-synthesis of qualitative studies according to JBI guidelines. CINAHL, EMBASE, MEDLINE, LILACS, PSYCinfo, Scopus and Web of Science databases were used. The findings were classified according to the level of reliability and credibility and categorized by similarity between contents.

**Results:**

229 articles were identified, and five were included in the final sample and organized into the categories: Emotional impact; and Understanding nursing role. The level of evidence found was moderate.

**Final considerations:**

nursing experiences are permeated by emotional exhaustion, feelings of frustration and difficulty in understanding their professional role. The need for training spaces that qualify this care is highlighted.

## RESUMO

**Objetivo:**

sintetizar as evidências de estudos qualitativos sobre as experiências da enfermagem frente ao cuidado em saúde mental infantojuvenil em serviços especializados.

**Método:**

revisão sistemática, com metassíntese de estudos qualitativos, segundo as diretrizes do JBI. Utilizaram-se as bases de dados CINAHL, EMBASE, MEDLINE, LILACS, PSYCinfo, Scopus e *Web of Science*. Os achados foram classificados segundo o nível de confiabilidade e credibilidade, e categorizados pela similaridade entre os conteúdos.

**Resultados:**

229 artigos foram identificados, e cinco foram incluídos na amostra final e organizados nas categorias: Impacto emocional; e Compreensão do papel da enfermagem. O nível de evidência encontrado foi moderado.

**Considerações finais:**

as experiências da enfermagem são permeadas por desgaste emocional, sentimento de frustração e dificuldade de compreender o seu lugar profissional. Destaca-se a necessidade de espaços formativos que qualifiquem este cuidado.

## RESUMEN

**Objetivo:**

sintetizar evidencia de estudios cualitativos sobre experiencias de enfermería en la atención a la salud mental de niños y adolescentes en servicios especializados.

**Método:**

revisión sistemática, con metasíntesis de estudios cualitativos, según directrices del JBI. Se utilizaron las bases de datos CINAHL, EMBASE, MEDLINE, LILACS, PSYCinfo, Scopus y Web of Science. Los hallazgos se clasificaron según el nivel de confiabilidad y credibilidad, y se categorizaron según la similitud entre los contenidos.

**Resultados:**

se identificaron 229 artículos, cinco de los cuales fueron incluidos en la muestra final y organizados en categorías: Impacto emocional; y Comprender el papel de la enfermería. El nivel de evidencia encontrado fue moderado.

**Consideraciones finales:**

las experiencias de enfermería están permeadas por agotamiento emocional, sentimientos de frustración y dificultad para comprender su lugar profesional. Se destaca la necesidad de espacios de formación que cualifiquen esta atención.

## INTRODUCTION

Child and adolescent mental health has been a topic of great interest in recent years. According to the report on the State of the World’s Children, in Latin America and the Caribbean, around 16 million adolescents between the ages of 10 and 19 experience a mental health problem. The most common problems are depression, suicide and self-injury^(1)^. In addition to intense individual and family suffering, these problems generate socioeconomic, cognitive, emotional damage, even disability or premature death of children and adolescents with a psychiatric diagnosis^(1)^. The World Health Organization (WHO) in partnership with the United Nations Fund (UNICEF) has organized meetings as a strategy to face this problem^(1)^.

In this context, specialized services increasingly occupy a prominent role with high demand for the “resolution” of these problems. These spaces are configured as one of the possibilities of care and are present in several places in the world, offering care in a hospital and/or community context to children and adolescents who have some alteration in behavior and/or development^(2, 3)^.

The importance of nursing in the daily life of these services is known and studied at a global level. These professionals play a therapeutic role in welcoming, listening, support for developing relational, emotional and social skills, in addition to encouraging the construction of independence and trust, and may be leading actors in constructing emancipatory care plans. At the same time, authors point out that when their actions are supported by the biomedical paradigm with the logic of screening, diagnosis and consequent medicalization, they prioritize controlling behaviors and silencing “symptoms” to the detriment of person-centered care^(4, 5, 6)^.

The medical influence on nursing practices is one of the factors that make it difficult to break with the biomedical paradigm, as it comes from a historical construction of the health-disease process, which can reinforce the fragility and uncertainty of professionals in recognizing their therapeutic possibilities in mental health and in the psychosocial field^(6)^. Thus, it is understood that nursing may or may not favor depathologizing therapeutic processes.

The need to train and qualify the actions of these professionals in this field has been pointed out^(6, 7)^, and theconcept of experience can subsidize the analysis of this problem. The etymology of the word “experience” comes from Latin *experientia.ae.* One of the definitions tells us that “all knowledge acquired through the use of the senses” can be called experience^(8)^. In this way, the experience reflects the affections that arise from the relationships between nursing professionals and children and adolescents. Recognizing them can be an important strategy to highlight the gaps and guide the direction of this action.

Thus, this study asked: what is the evidence on nursing experiences regarding child and adolescent care in services specialized in child and adolescent mental health? A previous search in MEDLINE, Cochrane, CINAHL, PROSPERO and JBI databases and registries did not find protocols or reviews that answered this question.

## OBJECTIVE

To synthesize evidence from qualitative studies on nursing experiences regarding child and adolescent mental health care in specialized services.

## METHODS

### Study design

This is a systematic review with meta-synthesis of qualitative studies conducted according to JBI guidelines^(9)^ according to an *a priori* protocol registered on the PROSPERO platform number CRD42022297605^(10)^. Any protocol deviations were identified and reported in the present study.

### Study selection

The selection criteria were supported from research question formulation that was carried out through the PICo strategy. P (population) refers to nursing professionals who make up care teams in services specialized in child and adolescent mental health. Studies carried out with nursing students were excluded. I (interest) refers to the experience of nursing work. Co (context) refers to specialized services in child and adolescent mental health, such as the Psychosocial Care Center for children and adolescents (CAPSij - *Centro de Atenção Psicossocial infantojuvenil*), mental health clinic and psychiatric hospital for children and adolescents. The combination of descriptors is presented in the supplementary material.

Qualitative studies were considered, including, but not limited to, phenomenological studies, Grounded Theory, ethnography, descriptive qualitative, action research. There was no time and language limitation. Literature reviews, systematic reviews, case studies, editorials and unpublished studies (dissertations, theses, papers presented at events, etc.) were excluded.

### Data collection

Data collection was conducted in seven electronic databases, such as MEDLINE, LILACS, EMBASE, PSYCinfo, Web of Science, CINAHL and Scopus. For each base, specific descriptors were used, selected by Medical Subjects Headings (MESH) and Health Sciences Descriptors (DeCS): Mental Health Services; Mental Health Care; Psychiatric Services; Job Experience; Care Experience; Child Health; Adolescent Health; Nurse; Nursing.

Rayyan was used, which favors organization and accuracy in study selection^(11)^. After this step, the articles were read in full to define the final sample. The references of the studies included in this step were analyzed. Any disagreements that arose among reviewers were resolved through discussion, or with a third reviewer^(12)^.

The selection process followed the Preferred Reporting Items for Systematic Review in Meta-Analyses (PRISMA 2020) flowchart^(13)^. Data were extracted by two reviewers using the JBI qualitative evidence tool for data extraction on the SUMARI platform^(9)^ containing details of population, context, geographic location, study method and phenomenon of interest. The extraction of discoveries occurred in two ways, at the level of the theme^(14, 15, 16)^ and sub-theme^(17, 18)^, contained in the selected articles, and each discovery was extracted accompanied by an illustration, that is, a speech of the subjects who participated in the referred researches. The review team analyzed each extracted finding and grouped them into categories based on the similarity in meaning of each of them. To facilitate visualization, this was initially described on sheets of paper and each reviewer analyzed the similarities of meanings separately, then a joint comparison work was carried out to reach consensus. After these moments, the discoveries were included in SUMARI, making it possible to carry out the synthesis process^(9)^.

### Data analysis and treatment

All findings were classified according to the JBI Credibility Levels as “unequivocal”, “credible”, or “unsupported”. Both reviewers assessed the extracted findings and reached agreement on their assigned levels of credibility. Findings accompanied by an illustration that is beyond any reasonable doubt and therefore not open to challenge were assessed as “unequivocal”^(19)^. Findings assessed as “credible” corresponded to those whose findings are accompanied by an illustration without a clear association and, therefore, open to challenge. “Unsupported” findings were those whose findings are not supported by the data. Only findings classified as “unequivocal” or “credible” were included in aggregation, according to the JBI approach^(19)^. From the classifications of findings and illustrations, reviewers created categories that together produced a single comprehensive set of findings. The category grouping process generates a meta-aggregation that is presented in a descriptive summary format^(19)^.

The studies were assessed by two independent reviewers using the standardized instrument of the JBI critical Appraisal Skills Program Qualitative Research Checklist^(20, 21)^. This process was carried out on the SUMARI platform^(9)^, and all studies were included independently in the answers to each question. The result was used to support ConQual Table with a summary of the findings as well as in the review discussion stage and limitations. Any disagreements that arose among reviewers were resolved through discussion, or with a third reviewer. To assess the reliability of synthesized qualitative findings, synthesized findings were classified according to the ConQual approach^(19)^.

Each article is initially ranked high, moderate, low, and very low – qualitative articles are initially ranked high. From this starting point, they are assessed in terms of reliability and credibility^(19)^. Reliability is based on the first five questions of the critical assessment instrument for qualitative studies. These questions are related to the suitability of conducting the research with its objectives and purposes. Depending on the number of “yes” answers to questions 1 to 5, ranking per article goes up or down (or remains the same): i) 4-5 “yes” answers, rating remains unchanged; ii) 2-3 “yes” answers, assessment goes down 1 level; iii) 0-1 “yes” answers, assessment goes down 2 levels^(20, 21)^. For the credibility score, reviewers seek to identify the level assigned to the findings that were synthesized, verifying how many findings and which level was assigned. For each finding, reviewers assigned one of the levels: Unequivocal (U); Credible (C); Unsupported (US). The ranking process considers the following score calculation for each synthesized finding: i) All unequivocal findings: the grade remains unchanged; ii) Combination of unequivocal/credible findings: one level is downgraded (-1); iii) Credible/Unsupported findings: downgraded three (-3)^(19)^. This rating provided a “moderate” degree of confidence for the synthesized finding due to reliability issues (most studies had no statement of researcher location and no acknowledgment of author influence on research).

## RESULTS

In the six searched databases, 229 studies were identified. After assessing the titles and abstracts, according to the eligibility criteria, 29 were selected for full reading and 24 were excluded. It is noteworthy that the two reviewers resolved the differences between five articles, not requiring the presence of a third reviewer to reach a consensus. Thus, five studies were part of the final sample.

**Figure 1:**
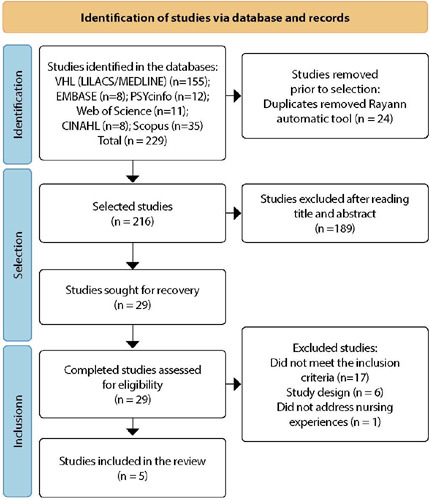
Flowchart of the review article selection process according to PRISMA, 2020

In Figure 1, it is presented the flowchart with the path followed for study selection according to the PRISMA recommendation^(13)^.

Caption: VHL - Virtual Health Library; LILACS - Latin American and Caribbean Literature in Health Sciences; MEDLINE - Medical Literature Search and Analysis System Online; CINAHL - Cumulative Index to Nursing and Allied Health Literature.

### Methodological quality assessment

The results of each of the included studies and their critical assessment are expressed in Chart 1. Questions related to congruence between methodology, objectives, methods, representation and analysis of results were fully contemplated. We observed some limitations related to the researcher’s theoretical and cultural location, his influence on the research and lack of evidence from ethics committee.

**Chart 1:** Critical assessment of eligible studies

Quote	Q1	Q2	Q3	Q4	Q5	Q6	Q7	Q8	Q9	Q10
Baldwin L, 2002^(16)^	Y	Y	Y	Y	Y	Y	Y	Y	No	Y
Anderson M. *et al*., 2003^(15)^	Y	Y	Y	Y	Y	U	U	Y	U	Y
Reiss R. *et al*., 2015^(14)^	Y	Y	Y	Y	Y	Y	Y	Y	Y	Y
Wilson RL, Usher K, 2015^(18)^	Y	Y	Y	Y	Y	U	Y	U	Y	Y
Bertram JE, Sarah CN, McMillen JC, 2013^(17)^	Y	Y	U	Y	Y	U	No	Y	Y	Y
%	100.0	100.0	80.0	100.0	100.0	40.0	60.0	80.0	60.0	100.0

*Y = Yes, U = Unclear, N = No; Q1. Is there congruence between the declared philosophical perspective and the research methodology? Q2. Is there congruence between the research methodology and the research question or objectives? Q3. Is there congruence between the research methodology and the methods used to collect the data? Q4. Is there congruence between the research methodology and representation and data analysis? Q5. Is there congruence between the research methodology and interpretation of results? Q6. Is there a statement locating the researcher culturally or theoretically? Q7. Is the influence of the researcher on the research and vice versa addressed? Q8. Are participants and their voices adequately represented? Q9. Is the research ethical according to current criteria or, for recent studies, is there evidence of ethical approval by an appropriate body? Q10. Do the conclusions drawn in the research report arise from data analysis or interpretation?*

### Characteristics of selected studies

The studies included in the sample were carried out between 2002 and 2015, one in Australia^(18)^, one in the United States^(17)^ and three in the United Kingdom^(14, 15, 16)^. The total sample of professionals surveyed was 61 nurses.

Three studies had as a phenomenon of interest nurses’ performance in multidisciplinary teams of specialized services in child and adolescent mental health^(16, 17, 18)^ and the other two^(14, 15)^,on the specific nursing child and adolescent care with diagnoses (suicide and borderline personality disorder). One of the studies brought the voice of other professionals from the multidisciplinary team, in addition to nurses. However, it was assessed by the reviewers and, given the significant participation of nurses, it was included in the sample.

Semi-structured interview was used by researchers in four studies and one used a structured questionnaire. Chart 2 details the characteristics of studies.

**Chart 2:** Characteristics of included studies

Study	Data collection and analysis methods	Country	Phenomenon of interest	Context	Population	Description of main results
Baldwin L, 2002^(16)^	Semi-structured interview collection method/thematic analysis method	United Kingdom	Perception of nurses’ role in child and adolescent mental health teams.	Outpatient mental health service for children and adolescents.	Outpatient mental health service for children and adolescents	While other areas were able to define what was unique to their role, it was found that there was no consensus among nurses about their role due to their training in nursing. There is a risk that, and nurses cannot develop a clearer rationale for their role, or be better able to articulate what they do in these teams, that role may be lost.
Anderson *et al*. 2003^(15)^	Collection method was semi-structured interview and structured questionnaire/analysis method was the Grounded Theory	United Kingdom	Nursing professionals’ and physicians’ perceptions on suicidal behavior among young people.	Pediatric medicine, accident and emergency services, and psychiatry.	Total 45, with 28 nurses	Two main categories and associated subcategories were identified: Experiences of frustration in practice (subcategories: Non-therapeutic situations; Insubstantiality of interventions and value of life) and relationship strategies with young people (subcategories: Specialized competences in care; Reflections on one’s own experience). The meanings of these categories highlight barriers in the relationship between nurses and physicians that they have with young people who engage in suicidal behavior. If suicide prevention policies around the world are to succeed, the phenomena that impact communication between these professionals and young people need to be addressed in research, education and practice development.
Reiss et al. 2015^(14)^	Semi-structured interview collection method/interpretive phenomenological analysis method	United Kingdom	Nurses’ experience in the specific care for children and young people with personality disorders.	Inpatient unit of mental health services for children and adolescents.	Total 6 nurses	We found two themes. One on the emotional impact of working with these young people and the other on the ways it has affected the clinical team dynamic. Many of the participants’ experiences and desires, such as anger, feeling awkward, and wanting training, are similar to those of nurses working with adults with Parkinson’s disease. Others seem to be more pronounced in this scenario, particularly in relation to the lack of diagnostic certainty, which involves the dilemmas that this brings in the provision of a service, and the difficult interpersonal dynamics in the nurse-patient relationship.
Wilson RL, Usher K,^(18)^	Mixed methods; data collection with in-depth interviews. Data were entered into nVivo10 (QSR International, Doncaster, Australia) and analyzed using thematic techniques.	Australia	Actions that help young people with early mental health issues.	Rural health service.	81 respondents, with 20 nurses	Research findings indicate that providing positive mental health first encounters to rural youth in their communities enables successful initial and ongoing mental health help.
Bertram JE, Sarah CN, McMillen JC, 2013^(17)^	Qualitative, descriptive study; semi-structured interview script.	United States	Describe the details of psychiatric nurses’ work in a multidisciplinary mental health team and identify gaps.	Care service for children and adolescents with mental health problems.	01 nurse	Psychiatric nurses’ role filled several necessary gaps, as she was able to provide a keen eye for processes and patterns related to psychiatric diagnosis and prescription, help to bolster the confidence of case managers, youth and foster parents to advocate for youth mental health care and provide an integrated perspective on physical and mental health for a population with complex comorbidities.

### Review findings

**Figure 2:**
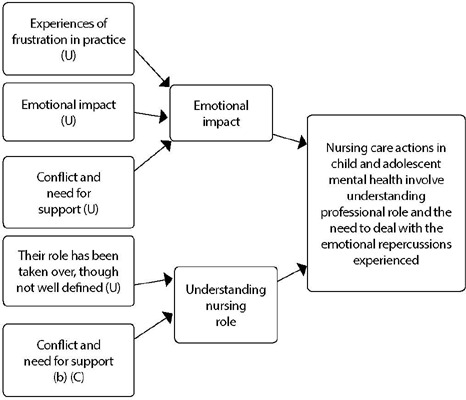
Meta-aggregation and meta- synthesis result in which U – Unequivocal and C – Credible

Five qualitative findings were extracted. Of these, four findings were assessed as unequivocal and one as credible. The set of findings was grouped into two categories based on differences, similarities or complementarities. Figure 2 brings the grouping of categories that generated the following summary: nursing care actions in child and adolescent mental health involve understanding the professional role and the need to deal with the emotional repercussions experienced. It was considered essential to improve skills that favor effective communication, such as listening in care and team relationships.

Study analysis resulted in two main categories that supported the construction of meta-aggregation and will be explored below, namely: Emotional impact; Understanding nursing role.

### Emotional impact

The emotional impact category was created from four illustrations that addressed the emotional intensity of working with these adolescents and how this affects the care process in the team. The emotional responses reported by nurses, as in the following example, express a polarization of emotions ranging from intense anger to more cordial feelings.

*From frustration to happiness, annoyance and sadness, they tend to bring out the best and worst in you* ^(14)^. (U)

*You often find yourself in conflict with the decision makers. So asking a question can be experienced as an accusation, so you end up having an argument or an impasse and then the whole point is lost, so I think reflective questioning, reflective engagement has been something to do* ^(14)^. (U)

The feeling of frustration and the need for support were the main concepts associated with the professionals’ experiences.

*I think it’s frustrating because whatever support they’re getting doesn’t seem to be adequate, maybe* [...] *you can give them more information and they still go and do these things. It’s frustrating that we can’t help them or we can’t do something to stop them from doing that and maybe leading a more normal life* ^(15)^. (U)

*I think it would be nice to have, say, more training. I think that, just for us, let’s say, for the nursing team* [...] *there is not much focus on, let’s say, the diagnosis of personality disorder, for example* ^(14)^. (U)

### Understanding nursing role

The second category was created from five illustrations that are related to understanding nursing role as part of the multi-disciplinary team.

The first highlighted point is related to the type of demand received by specialized services in child and adolescent mental health, nursing area and care model specificities.

*Because I wanted to be a nurse for her and I also found it difficult, because she didn’t need nursing much, she needed therapy, so that’s when I found it very difficult to work with her because I really didn’t know what my role was in that* ^(14)^. (U)

*It’s a completely different kind of nursing in that regard, because you can’t put a bandage on it* ^(15)^. (U)

Together, an illustration depicts that the population is unaware of the therapeutic role that nurses play in mental health.

*We just don’t get those demands... I’ve spoken to many mothers of clients who just don’t know who to turn to* ^(18)^. (C)

Another component observed in the illustrations is that nursing professionals’ performance was associated with a generic role conditioned to the presence of a physician and consequent actions focused on diagnosis and drug treatment.

*It has always been like that. Where there are physicians, there are nurses* ^(16)^. (U)

*The perspectives of multiple informants helped the nurse to distinguish which diagnoses were accurate or useful in the present and which could be eliminated because they were no longer valid. The diagnostic review was consolidated in summary form into a diagnostic and medication summary document* ^(17)^. (U)

Chart 3 summarizes the findings with the Score ConQual.

**Chart 3:** ConQual summary of findings

Title: Nursing experience in child and adolescent mental health services: a qualitative systematic review				
Participants: nursing professionals Phenomenon of interest: experience regarding child and adolescent mental health Context: specialized services in child and adolescent mental health				
Summary of findings	Search type	reliability	Credibility	Score ConQual
Nursing care actions in child and adolescent mental health involve understanding the professional role and the need to deal with the emotional repercussions experienced	Qualitative	High*	Moderate^†^	Moderate 4(U) + 1(C)

*4-5 “yes” answers, finding remains unchanged; ^†^The combination of unequivocal/credible findings downgraded one notch (-1)

According to the chart above, the synthesis of the findings presents high reliability, since the result of study quality assessment showed a yes answer for more than 4 criteria. However, credibility was assessed as moderate, since illustrations were classified as unequivocal and credible, i.e., not all of them significantly represented the finding presented in the primary studies.

## DISCUSSION

This systematic review with meta-synthesis of qualitative studies was developed to synthesize the evidence on nursing experiences regarding child and adolescent mental health care in specialized services. The synthesis revealed gaps related to the role that nursing has in this field of care and the emotional implications caused by it.

It is noteworthy that no study included was developed in Brazil, which is relevant, as the location of studies can emphasize the impact of goals established in the International Declaration on Youth Mental Health, an achievement of mental health workers in Australia, United Kingdom and Ireland, who claimed, for three years, improvements in the organization of services and new ways of caring for child and adolescent mental health. In this process, there was a highlight for nursing, which took over the role of consulting in child and adolescent mental health. This new role was created to take over the leadership of specialized services and also be present in schools, supporting mental health promotion and care actions, which justifies studies on nursing in this region^(22, 23)^.

Studies^(5, 24, 25)^ point out that the history of nursing in mental health care is a process in constant construction and is linked to the identification of madness by medicine. Even before the creation of large psychiatric hospitals, nursing already carried out the care for people with mental suffering in the wards of general hospitals. However, nursing finds it difficult to break with the biomedical paradigm and recognize its therapeutic function as part of the multidisciplinary team in mental health services. It is understood that the inheritance of the asylum model in which nursing role was subordinated to the figure of medical professionals and had the duty to respect and perform orders related to hygiene, food, medication, surveillance and discipline of hospitalized people is related to this difficulty in understanding their autonomous and therapeutic role^(24, 25)^.

Two of the studies included in the review^(17, 18)^ draw the attention of nurses to be concerned about society’s failure to recognize their value and role in mental health services. They point out that the potential of care centered on the subject, the Theory of Interpersonal Relationships and the comprehensive approach should be emphasized as a specific contribution of nursing in the context of specialized services, which would favor understanding regarding the presence of this category in care practice. The fact of not locating their contribution favors the feeling of not belonging to the nursing area and devaluation of their participation in the team. In this context, they suggest that it is urgent to understand and affirm this specific role.

These findings corroborate the study by Delfini, Toledo and Garcia (2021), which aimed to understand the nursing team’s (assistants, technicians and nurses) work process in a CAPSij, and concludes that this is supported by the lack of knowledge related to discrepancy between knowledge acquired in training and knowledge necessary for this care practice. They state that these professionals depend on the direction of other multidis-ciplinary team members, and place themselves in co-therapists’ role in child and adolescent mental health, basing the assistance from the medical diagnosis. Concomitantly, the authors point to a contradiction, since, despite the general basis of nursing care being relational, in mental health practice, these professionals are unable to appropriate this therapeutic action and live in tension about their role^(26)^.

In opposition to this, the study by Elias, Tavares and Munis (2020), which investigated nurses at a psychiatric hospital in relation to adult care, found that most consider themselves therapists due to the mental health actions they develop, such as listening, welcoming, bonding and, above all, the appropriation of the Theory of Interpersonal Relations, emphasizing that awareness of being therapeutic is essential for this process to be established. It is worth noting that most nurses had postgraduate training in the field of mental health^(6)^.

It is interesting to note that the hospital environment, apparently, favors the understanding of nursing role in mental health, since, in the psychosocial paradigm, care actions are expanded and, therefore, the difficulty may be inherent to this complex network of extramural roles and functions within the multidis-ciplinary team. This is even more specific and complex in child and adolescent care, as pointed out by the studies included in this review.

In this sense, there is a consensus among the authors on the importance of adapting nursing team education and training so that the role in the mental health child and adolescent care is understood considering the current models of care^(6, 14, 18, 25, 26)^. Although nursing is often given meaning based on the performance of concrete procedures and techniques, the relational elements present since their training make it a potential actor of change, enabling its therapeutic professional performance^(6)^.

With a view to investing in the promotion of person-centered and rights-based approaches to community mental health services, the Pan American Health Organization has encouraged a set of good practices in mental health. Peer support is one of them, and provides for individual or collective sessions mediated by people who have similar experiences. With the experience, the connection between these people gains other meanings and access to current suffering, which favors the consolidation of support networks, which traditional models find difficult to achieve^(27)^. This is an example of a practice that can be used by nurses as a strategy to qualify their care actions in the psychosocial field.

The second category defined by the findings of this review was the emotional impact, similar to the scientific literature when identifying the polarization of feelings and emotions in the mental health child and adolescent care.

A study that assessed multidisciplinary team practices aimed at adolescents in a CAPSij showed that work in this context varies between pleasure and suffering. It points to the motivation of professionals regarding the possibilities of care in this phase, envisioning concrete transformations in the lives of young people, at the same time as they feel powerless to change some realities, especially when they involve situations of vulnerability, violence and/or neglect, which generate frequent feelings of frustration^(28)^.

The fact that children and adolescents spend most of their time with nursing professionals is often cited, as it is understood that this contact favors the emotional intensity of this category, when compared to the others, who relate for a shorter time. Caring for this group, when in psychological distress and/or with mental health problems, causes emotional experiences that nursing professionals are not always prepared to deal with, as managing the feelings that permeate these situations and also promoting affective care relationships is particularly exhausting^(29, 30)^.

In view of this, a resource pointed out by one of the studies included in the review to dilute the emotional impact was the clinical supervision space, which needs to have flexible hours to favor the participation of nurses who are present at different times in services^(14)^. In this sense, we understand that supervisory spaces as well as training spaces are resources that can be powerful in offering support and increasing knowledge about nursing role in this field, which may minimize uncertainties in relation to these professionals’ therapeutic role in their daily practices.

No studies were found that exclusively addressed the emotional effects on nursing professionals, and, for this reason, the discussion used studies that addressed these effects in child and adolescent care in pediatrics.

### Study limitations

The limitations of this review are related to the restricted sample of eligible studies and the location of research, which may be related to scenario delimitation to specialized services in child and adolescent mental health, since they are organized according to local policies, and the actions of nursing in this field also happen in other care settings. These limitations reinforce existing gaps regarding this topic and the consequent need to carry out new studies in different contexts. Most of studies included in the review did not talk about the researcher’s influence on the research and vice versa, and this was not mentioned. One of the studies did not bring many illustrations to support the findings (themes or subthemes), which made it difficult to extract a larger amount of data and create categories.

### Contributions to nursing

The evidence found in this review allowed us to understand that it is necessary to guarantee training spaces that go beyond traditional training still based on biomedical/psychiatric nursing, in addition to spaces that welcome nursing professionals, favoring listening and presenting new psychosocial modes of mental health child and adolescent care based on the challenges experienced by these professionals in their daily meetings. In this regard, we recommend: ensuring reflective spaces for education, training and improvement of practices in specialized services in child and adolescent mental health; using theoretical resources available in mental health nursing, such as the Theory of Interpersonal Relationships; including theoretical skills and emotional skills related to trust in carrying out care practices and contributions to the multidisciplinary team in professional training curricula.

## FINAL CONSIDERATIONS

This systematic review with meta-synthesis found that evidence from qualitative studies on nursing professionals’ experiences in providing care in specialized mental health services for children and adolescents reveal emotional exhaustion, feelings of frustration and inability to understand their place as part of a multidisciplinary team. These findings confirm the need to welcome and qualify these experiences. Training spaces developed from the daily routine of nursing care in these services and that also offer listening to the emotional impacts inherent in these encounters can be promising.
